# A study on the potential role of autophagy‐related protein 10 as a biomarker for ulcerative colitis

**DOI:** 10.14814/phy2.14825

**Published:** 2021-04-27

**Authors:** Fatemeh Abbasi Teshnizi, Nasrin Kazemipour, Saeed Nazifi, Kamran Bagheri Lankarani, Masood Sepehrimanesh, Iman Razeghian Jahromi

**Affiliations:** ^1^ Biochemistry Division Department of Basic Sciences School of Veterinary Medicine Shiraz University Shiraz Iran; ^2^ Clinical Pathology Division Department of Clinical Sciences School of Veterinary Medicine Shiraz University Shiraz Iran; ^3^ Health Policy Research Center Shiraz University of Medical Sciences Shiraz Iran; ^4^ Department of Biology University of Louisiana at Lafayette Louisiana at Lafayette LA USA; ^5^ Cardiovascular Research Center Shiraz University of Medical Sciences Shiraz Iran

**Keywords:** ATG10, inflammatory bowel diseases, miR‐519a, ulcerative colitis

## Abstract

**Purpose:**

Ulcerative colitis (UC) is a lifelong disease with unclear etiology and increasing prevalence worldwide. Autophagy has been reported to play roles in the pathogenesis and progression of UC. Here, we aimed to analyze the expression of autophagy related protein 10 (ATG10) and its regulator, micro‐RNA (miR) 519a, in UC patients.

**Methods:**

The level of ATG10 in the serum, stool, and colon biopsies from 15 UC patients and 30 non‐UC healthy individuals (HC) group was measured by ELISA. Also, the blood level of miR‐519a was investigated by quantitative real‐time PCR.

**Results:**

We found 13.63 ng/ml versus 0.99 ng/ml, 11.01 ng/ml versus 1.11 ng/ml and 6.41 ng/ml versus 3.21 ng/ml of ATG10 in the stool, colon tissue, and serum of UC and HC, respectively. There was no significant difference in the expression of miR‐519a in the blood samples of UC and HC.

**Conclusions:**

ATG10 might be a potential non‐invasive diagnostic biomarker for UC.

**Main Points**

- Comparison between ulcerative colitis patients and control group regarding ATG10and miR‐519a was done.
- Patients with UC showed higher content of ATG10 in serum, stool and colon biopsy compared to the control group.
- Insignificant higher content of blood miR‐519a in the HC group compared to the UC group.

## 1 INTRODUCTION

Ulcerative colitis (UC) and Crohn's disease (CD), classified as inflammatory bowel diseases (IBD), are lifelong diseases, arise from an interaction between genetic and environmental factors, and are predominantly observed in developed countries (Hosseini et al., 2016). They are increasing worldwide with the increasing pace of the westernization of societies. IBD is often diagnosed during the most productive years of adulthood and can severely impact all aspects of a patient's life (Kim et al., 2014). Some studies have reported that patients with UC are at increased risk of developing colorectal cancer (CRC; Levy et al., 2017). The precise etiology of IBD is unknown; therefore, curative medical therapy is not yet available for this disease (Nielce et al., 2018).

Autophagy is an evolutionarily conserved catabolic pathway that occupies a central role in the biology of most eukaryotes, the regulation of core metabolism, damage control, and cell death (Galluzzi & Green, 2019). The role of autophagy in the pathogenesis and progression of IBD has been studied more recently. Defects in this pathway have been implicated in IBD (Nielce et al., 2018). Several autophagy‐related genes are associated with various pathologies of IBD and play important roles in immune cells, such as macrophages and dendritic cells, as well as intestinal epithelial cells, including Paneth cells (Iida et al., 2019). Autophagy affects the pathogenesis of IBD through various pathways, such as the regulation of pathogen clearance (Gutierrez et al., 2007; Rich et al., 2003) and modulation of the immune response (Iida et al., 2019). Evidence has shown that impaired autophagy disturbs the function of the intestinal barrier, influences the immune responses, endoplasmic reticulum (ER) stress, and ROS production, causes an abnormal inflammatory reaction and promotes the occurrence and development of IBD (Hosomi et al., 2015, Randall‐Demllo et al., 2013, van de Veerdonk & Dinarello, 2014, Wallace et al., 2014). Autophagy‐related proteins (ATGs) play an integral role in the complex cellular processes of autophagy (Levy et al., 2017). ATG10, as an E2‐like enzyme, is essential for autophagy since it promotes ATG5‐ ATG12 complex formation (Kongara & Karantza, 2012), which mediates the autophagosome formation (Kongara & Karantza, 2012). Mounting evidence suggests that miRNAs can function as autophagy regulators and play an important role in IBD. MicroRNAs (miRNAs) are endogenous non‐coding small RNAs and act as post‐transcriptional regulators of gene expression. Research indicates that microRNAs regulate autophagy via different pathways, play a critical role in the IBD process, and provide a new perspective for IBD research (Wang et al., 2018). Although there has been great progress in the development of therapeutic strategies targeting autophagy in intestinal diseases, the role of autophagy in the pathogenesis of IBD has not been fully clarified (Wu et al., 2019). There are numerous studies on knockout and knockdown models for evaluating the effects of different ATG proteins on IBD, but there is a lack of in vivo studies on human UC. In addition, the distribution of autophagy‐related proteins and their fecal excretion are unknown in human UC.

Therefore, we aimed to compare the expression levels of ATG10 in the serum, stool, and colon biopsies from healthy individuals and UC patients. We hypothesized that the expression level of ATG10, as a possible potential biomarker, would be distributed differently in different samples from UC patients versus the control group. Also, in this study, the expression levels of ATG10 regulatory microRNA, miR‐519a, in the blood sample of UC patients and non‐UC controls were measured.

## 2 MATERIALS AND METHODS

### 2.1 Study population

We included two groups of individuals including 15 UC patients and 30 healthy controls who attended to the IBD Centre of Faghihi and Mottahari hospitals from May 2017 to February 2018. Healthy controls were selected among the individuals complaining of some gastrointestinal symptoms which their health, then, was confirmed after colonoscopy, serology and histopathology. They were matched to the patients by age (UC, 43.96 ± 14.89 years (19–70 years), healthy control (HC): 49.5 ± 11.86 years (23–79 years)) and gender. The inclusion criteria were confirmed diagnosis of UC for the patient group and age 18–80 years. Patients with prior history of malignancy, use of some medications including NSAIDs and antibiotics, parasitic diseases, familial adenomatous polyposis, and other significant diseases that could possibly interfere with the study protocol were excluded. UC was strictly diagnosed by examination, colonoscopy, and histopathology. The study received the approval of the local ethics research committee (IRB No. IR.SUMS.REC. 1395.S1217) and all the study subjects gave their verbal and written informed consent before participation. The characteristics of the participants are provided in Table 1.

**TABLE 1:** The characteristics of the patients with ulcerative colitis (UC) and healthy control (HC)

Characteristics	UC	HC
Number	15	30
Sex (M:F)	6:9	13:17
Age (mean ± SD, range, years)	43.96 ± 14.89 (19–70)	49.5 ± 11.86 (23–79)
Weight (mean ± SD, range)	64.9 ± 10.96 (52–90)	70.32 ± 14.5 (54–100)
Disease duration (mean ± SD, range, years)	9.47 ± 5.72 (1–22)	NA
Disease intensity (%)		
Mild	33.3 (5/15)	
Moderate	53.3 (8/15)	
Severe	13.33 (2/15)	
Disease extent		
Proctitis	5 (33.4)	
Left‐sided colitis	10 (66.6)	

### 2.2 Sample preparations

Serum, stool, and colon biopsies were collected from the studied groups. Pinch biopsies from inflamed mucosa of colon regions with clear symptoms of UC such as erythema, the loss of vascular pattern, and ulceration or spontaneous bleeding were collected. The healthy controls pinch biopsies were taken from the same regions of the colon as the UC patients (mostly rectum). Tissue samples were homogenized by several freeze‐thaw cycles, followed by mechanical homogenization and exposure to ultrasonic waves (probe: 0.1 mm, 100 W, 30 kHz; UP100H Hielscher Ultrasonic GmbH). Tissue homogenates were then stored at −80°C for further analysis by ELISA. Stool samples were collected during the clinical examination of patients and healthy participants one day prior to colonoscopy procedure. One milliliter of PBS buffer was added to 100 mg stool samples regardless its texture, followed by vortexing for 15–20 min at 4°C until all the material was suspended. Afterward, the solids were separated by centrifugation for 10 min, 5000 *g* at 4°C and the extract was stored at −80°C until further assay.

Approximately, 5 ml of blood was collected and divided into two equal samples. The first 2.5 ml was utilized for serum isolation; the remaining was transferred into tubes, containing heparin as an anticoagulant, to be subsequently used for RNA extraction. Serum was isolated after centrifugation (1000 *g* for 20 min) and stored at −20°C for ELISA measurement of ATG10.

### 2.3 Sandwich ELISA assay

Once the serum, stool and tissue samples were prepared and homogenized, they were analyzed by sandwich ELISA using My BioSource human ubiquitin‐like‐conjugating Enzyme ATG10 ELISA Kit (MyBioSource). The ELISA plate was read at 450 nm using an ELISA reader (Convergys).

### 2.4 Quantitative real‐time PCR

Blood total RNA was extracted using Denazist total RNA isolation kit (Denazist) according to the manufacturer's instruction. cDNA was produced using a cDNA Synthesis kit (Pars Genome) and Veriti 96‐well Thermal Cycler. Moreover, SYBRGreen‐based quantitative real‐time PCR (qRT‐PCR) was used to examine the changes in the expression of miR‐519a in target groups. StepOne Real‐Time PCR System (Applied Biosystems) was used to perform real‐time PCR amplification. A thermocycler profile of 95°C for 10 min, followed by 40 cycles of 63.5°C for 2 s, and 72°C for 1.3 s, was used for the amplification of miR‐519a. U6 small RNA was used as an internal control and two replications were performed for each sample. Forward and reverse primers of miR‐519a were purchased from Pars Genome. The expression of miR‐519a was compared in controls and patients using the 2^−∆∆Ct^ method.

### 2.5 Statistical analysis

Quantitative and qualitative data were expressed as mean ±SD and frequency (percentage). To compare the ATG10 contents and expression level of miR‐519a, the data were analyzed by independent t‐test and Mann‐Whitney test using SPSS version 21. *p* < 0.05 was considered significant. Correlation between the ATG10 levels and disease magnitude was assessed by Spearman test.

## 3 RESULTS

**FIGURE 1:**
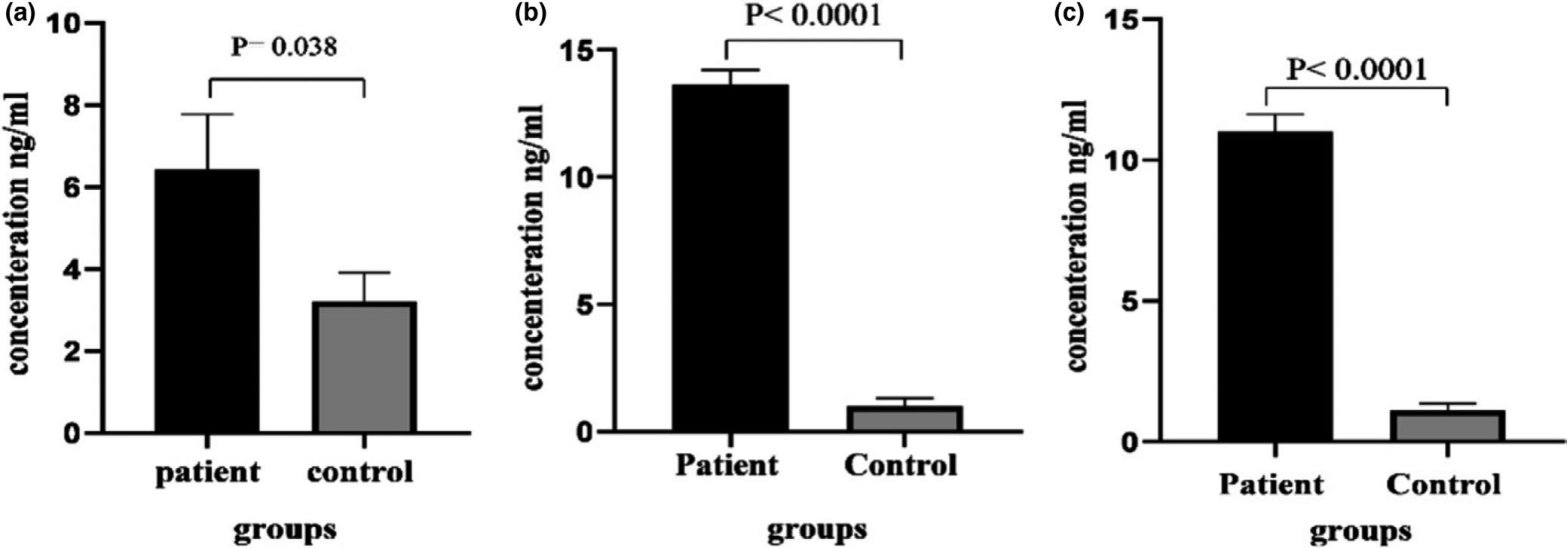
ATG10 concentration in UC and control groups measured by ELISA. The level of ATG10 in all samples was significantly higher in UC groups compared to that of the control group. ATG10 concentration in serum (a), stool (b), and colon biopsies (C)

The ATG10 level was significantly higher in the serum (6.41 ng/ml vs. 3.21 ng/ml, *p* = 0.038), stool (13.63 ng/ml vs. 0.99 ng/ml, *p* < 0.001) and colon biopsy (11.01 ng/ml vs. 1.11 ng/ml, *p* < 0.001) samples of UC patients compared to those in the control group (Figure 1).

**FIGURE 2:**
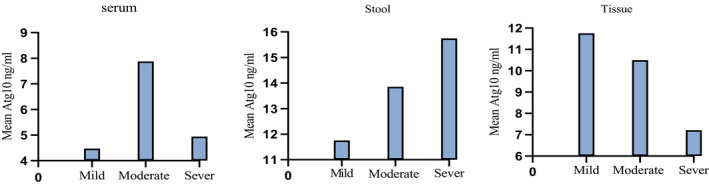
Mean Atg10 levels in patients according to the severity of the disease. The correlation between ATG10 level and UC severity was evaluated by spearman correlation test. There was no significant association between the UC severity and ATG10 level in serum (rs = +0.127, *p* = 0.91), stool (rs = +1, *p* = 0.33) or tissue (rs = −0.96, *p* = 0.159) of patients. Correlation is significant at the 0.05 level (2‐tailed)

The correlation between ATG10 level and UC severity was evaluated by spearman correlation test. There was no significant association between the UC severity and ATG10 level in serum (rs = +0.127, *p* = 0.91), stool (rs = +1, *p* = 0.33) or tissue (rs = −0.96P=0.159) of patients (Figure 2). Even though there was a positive linear correlation in stool samples and a negative linear correlation in tissue samples, but they were not statistically significant. Correlation is significant at the 0.05 level (2‐tailed). However, considering the small sample size, further investigations are required in order to have a more precise conclusion on the association of Atg10 level and disease severity.

**FIGURE 3:**
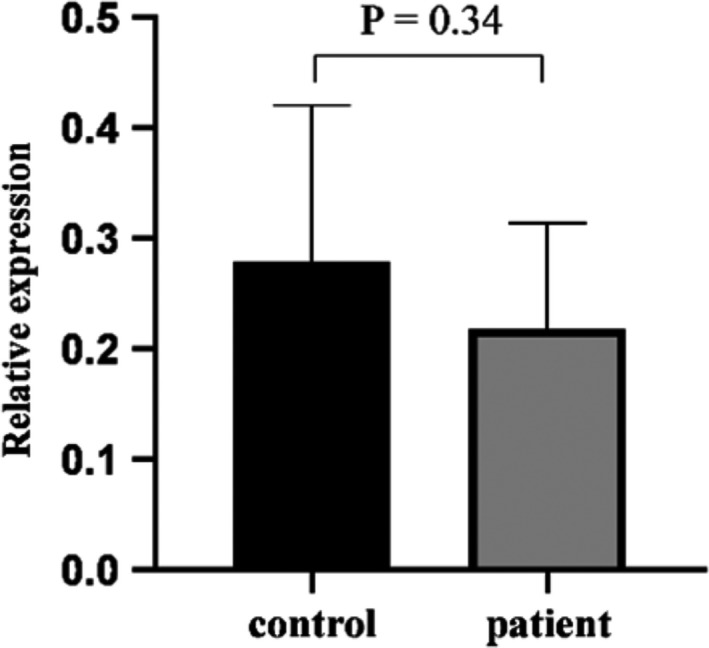
The expression of miR‐519a in the blood samples of UC and HC determined by qPCR. After the data were normalized with U6 (as an internal control), the expression of miR‐519a was calculated by fold change. Mann–Whitney test was carried out to analyze the data and the 2^−∆∆Ct^ method was utilized to compare the studied groups. There was no statically significant difference between the two groups regarding the expression of miR‐519a

The expression of miR‐519a in the blood samples of the studied groups was determined using qPCR. The data were normalized by U6 as internal control, and the expression levels for the two groups mentioned above were calculated using fold change. The expression of miR‐519a was higher in the HC group compared to that in the UC group, but the changes were not statistically significant (Figure 3, *p* > 0.05).

## 4 DISCUSSION

This study is the first human study that compared the serum, stool, and colon biopsies of UC patients and HC to examine whether there were any significant differences between the two groups regarding the ATG10 level. We showed that an increased level of ATG10 in UC patients may be due to accelerated autophagy in UC. However, we did not find any association between the ATG10 levels in different samples and the severity of the disease in our studied group. Considering the heterogeneous nature of UC and relatively small size of the studied group, further studies are needed to clarify any correlation between the severity of the disease and ATG levels. Many studies using conditional knockout mice have offered a deeper understanding of ATGs that affect intestinal inflammation and enteric pathogen infections during colitis. Various ATGs including ATG7, ATG16L1, leucine‐rich repeat kinase (LRRK), immunity‐related GTPase family M protein (IRGM), p62, optineurin (OPTN), and transcription factor EB (TFEB) play major roles in the maintenance of intestinal homeostasis (Kim et al., 2019). In addition, our group for the first time reported the association between lower serum levels of ATG7 and human UC (Lankarani et al., 2017). Also, we found a significant increased level of fecal ATG5 in UC patients (Ardali et al., 2020). So far, the importance of ATG10 in human UC has not been evaluated yet. ATG10 as an autophagic E2‐like enzyme plays a critical role in autophagosome formation by promoting ATG5‐ATG12 complex formation (Kongara & Karantza, 2012). Mounting evidence has shown that ATG10 had increased level of expression in malignancies such as CRC (Jo et al., 2017) and lung cancer (Xie et al., 2016). UC is a risk factor for CRC (Herszenyi et al., 2015), so ATG10 level in UC patients can be a good prognostic tool. A study showed that the expression of ATG10 increased in CRC and that increase was associated with lymph node metastasis and lymphovascular invasion. They suggested that ATG10 can be a potential prognostic biomarker in CRC (Jo et al., 2012). Similarly, the current study revealed that ATG10 increased in UC. An increased level of ATG10 in all samples of this study can suggest the upregulation of autophagy pathway in the colon. Also, it may reflect an interaction between ATG10 and other biological systems, such as ER stress response, which may have a significant impact on the pathogenesis of IBD (Hosomi et al., 2015).

The role of ATGs and the mechanisms conferring intestinal inflammation have not been fully clarified yet. Numerous genome‐wide association studies have confirmed that several autophagy‐related genetic variants, including ATG16L1 and IRGM, are closely associated with increased risk of IBD (Kim et al., 2019). Genetic association studies suggest that LRRK2/MUC19 and ATG7 deficiency aggravates intestinal inflammation in mouse models of colitis. Defective autophagy has been associated with the inflammasome activation, pyroptosis induction, and elevated susceptibility to colitis in mouse models (Pandey et al., 2019). On the contrary, and in agreement with our results, a study reported that the induction of autophagy could have negative effects, such as increased replication of intracellular pathogens and increased production of pro‐inflammatory cytokines, on the intestinal epithelial barrier function and play a conflicting role in some bowel diseases (Wu et al., 2019). Furthermore, in a recent study, two researchers reported that autophagy increased in dextran sulfate sodium (DSS)‐induced colitis in mice. They also observed upexpression of ATG5, LC‐3II, TNF‐α, IL‐6, IL‐17, and Beclin‐1 and down expression IL‐10 and Bcl‐2 in mice with colitis compared to those in normal mice. Accordingly, they speculated that the basal level of autophagy could protect the intestine from inflammation or injury (Chapman & Pekow, 2015). When intestinal inflammation occurs due to different reasons, such as bacterial overgrowth, excessive autophagy is induced, followed by a series of cascade‐like reactions, including enhanced pro‐inflammatory cytokine secretion, uncontrolled inflammation, cell death, and tissue injury. Based on this hypothesis that excessive autophagy can cause UC, it is reasonable to look for potential agents that can adjust autophagy to a reasonable level, which can, in turn, lead to further control over the cytokine network and attenuation of colon inflammation. Therefore, it can be concluded that autophagy activation can behave as a double‐edged sword in colitis and cancer. Autophagy should be controlled appropriately when facing stressful conditions or other predisposing factors of intestinal inflammation.

Despite numerous studies that have shown autophagy impairment can lead to IBD, in the present study, the upregulation of one autophagy protein, ATG10, was associated with active IBD. We assume that a basal level of autophagy and an appropriate amount of increase in the autophagy level is crucial for the prevention of UC; however, this assumption needs to be supported by further research. In general, some ATGs have non‐autophagic functions, some are not absolutely essential for autophagy, and some have both characteristics (Mizushima, 2018), so the level of ATGs can change independently of the level of autophagy. Thus, the increased level of ATG10 in this study may have not been caused by the increase in the autophagy level. For instance, host cells infection by different pathogens such as viruses and bacteria, which may affect the intestinal barrier and lead to UC, can be modulated by non‐autophagic functions of ATGs in membrane trafficking (Levine & Kroemer, 2019). Given the fact that autophagy is a complex and dynamic process, further investigation on other autophagy markers, such as LC3, P62, and Beclin‐1, can help us determine whether ATG10 changes are due to the changes in the autophagy level or other non‐autophagic pathways.

UC is one of the most important risk factors for CRC. Autophagy pathway is the main target of many recent studies for the development of effective CRC therapies (Xu et al., 2020). In agreement with our results, a study showed that ATG10 increased significantly in CRC and that increased expression of ATG10 was associated with lymphovascular invasion and lymph node metastasis. The study also introduced ATG10 as a potential prognostic marker in CRC (Jo et al., 2012). Considering the significant difference between UC patients and HC in terms of ATG10 levels, ATG10 can be suggested as a potential biomarker for UC. Since there was a need for some supportive biomarkers to this single protein, i.e., ATG10, the expression of miR‐519a in blood was measured, but there was no significant difference between the UC patients and control group regarding this microRNA. Although miR‐519a has been reported to have a regulatory effect on ATG10 (Su et al., 2015), no significant correlation was found between the miR‐519a expression and ATG10 levels in the present study. As key regulators of gene expression, miRNAs are involved in a broad range of biological pathways and widely dysregulated in inflammatory disorders (Lu et al., 2019). Several preclinical researches have supported the possibility that the alterations in miRNAs levels in inflammatory diseases can be reverted, which offers the promise of developing miRNA‐based therapeutics for inflammatory conditions in the future (Lu et al., 2019). miRNAs are markers of IBD and colon cancer and have the potential to be drug targets (Tili et al., 2017). Research indicate that miRNAs can regulate autophagy through distinct pathways and play a major role in the IBD development, thereby opening up a new avenue for IBD research. In the first study in this regard, differential expressions of miRNAs were evaluated in the intestinal tissue of UC patients (Wu et al., 2008). Xu et al. found a specific pattern for miRNAs expression in active UC tissues as follows: three miRNAs (miR‐375, miR‐422b, and miR‐192) were noticeably decreased and eight miRNAs (miR‐16, miR‐21, miR‐23a, miR‐24, miR‐29a, miR‐126, miR‐195, and let‐7f) were significantly increased(Xu & Zhang, 2016). Since the emergence of this pattern, further research studies have confirmed that miRNAs have an important role in the occurrence and development of IBD, even inactive UC, through the related inflammatory pathways, immune system, and other biological process. Also, recent findings suggests that miRNAs can act as autophagy regulators and play a key role in IBD by regulating different molecular pathways (Belcheva, 2017; Cao et al., 2017; Chapman & Pekow, 2015; Kalla et al., 2015; Murphy et al., 2012). A study showed that miR‐29a inhibited autophagy in intestinal epithelial cells in a mouse model of UC partly by decreasing ATG9A (Lin et al., 2018). miR‐20a was found to downregulate autophagy by targeting ATG16L1 in hypoxia‐induced osteoclast differentiation (Sun et al., 2015). miRNAs have been identified as new players in the pathogenesis of IBD and shown to regulate intestinal barrier integrity in UC (Kalla et al., 2015). miR‐143 was reported to induce intestinal inflammation by affecting autophagy and ATG2B, suggesting that miR‐143 might play a key role in the CD progression (Lin et al., 2018). A recent study has demonstrated that miR‐27b‐3p regulates the expression of ATG10 at the posttranscriptionally level in colorectal cancer cells in vitro and in vivo (Sun et al., 2020). miR 519a is considered to be a regulator of vesicle elongation in autophagy process via targeting Beclin 1, Atg 16 L1 and Atg10 (Huang et al., 2012). This miR has been assessed in several human cancers including gastric, ovarian (Tian et al., 2018) and glioma (Hong et al., 2016). In a recent study, the down‐regulation of this miR was associated with poor prognosis of gastric cancer. Given the inverse correlation between miR 519a expression and tumor differentiation and lymph node metastasis in gastric cancer patients, Cai et al., concluded that this miR might play a role in tumor progression. Considering its tumor suppressive characteristics in aforementioned cancers, miR 519a might be a promising prognostic biomarker (Cai et al., 2020) No previous study has examined the level of miR‐519a as an ATG10 regulatory microRNA, in UC patients. Our findings suggest that there is no statistically significant correlation between the blood levels of ATG10 and miR‐519a in patients and control group Further investigations on its expression in tissue and stool samples of the UC patients and healthy controls might provide us with new insights into its interplay between autophagy and CRC predisposition in UC patients.

## 5 CONCLUSIONS

It can be concluded that the onset of UC is associated with accelerated autophagy. In this study, we observed that the level of ATG10 was different between UC patients and HC. Given that many previous studies have reported some changes in the expression levels of ATG10 in CRC and documented its effect on tumor suppression and since UC has been shown to increase the risk of CRC (Desai et al., 2015), future studies are recommended to design a long‐term survey to follow up all UC patients and monitor changes in the levels of ATG10 or some other ATGs to identify potential prognostic markers for UC.

## CONFLICT OF INTEREST

To comply with the journal instructions, hereby, I confirm that this paper does not have any conflict of interest including any financial, personal, or other relationships with other people or organizations that could inappropriately influence, or be perceived to influence, their work.

## AUTHOR CONTRIBUTIONS

All authors contributed in all parts of study from designing the study to writing and preparing the manuscript. Masood Sepehrimanesh and Nasrin Kazemipour contributed to study design; Kamran Bagheri Lankarani and Fatemeh Abbasi Teshnizi and Saeed Nazifi contributed to performing the study, sampling and data collection. Fatemeh Abbasi Teshnizi, Saeed Nazifi, Nasrin Kazemipour and Iman Razeghian Jahromi helped in laboratory metabolites analysis and preparing the manuscript. All authors read and approved the final manuscript.

## ETHICAL APPROVAL

The study received the approval of the local ethics research committee (IRB No. IR.SUMS.REC. 1395.S1217) and all the study subjects gave their verbal and written informed consent before participation.
